# Identifying Key Factors Influencing Hospital Stay After Spine Surgery: A Comprehensive Predictive Model

**DOI:** 10.1177/21925682251331451

**Published:** 2025-04-01

**Authors:** Francesco Langella, Francesca Barile, Pablo Bellosta-Lòpez, Federico Fusini, Domenico Compagnone, Daniele Vanni, Marco Damilano, Pedro Berjano

**Affiliations:** 1 IRCCS Ospedale Galeazzi-Sant’Ambrogio, Milan, Italy; 2 Universidad San Jorge, Campus Universitario, Villanueva de Gállego, Zaragoza, Spain; 3 Department of Orthopaedic Surgery and Traumatology, Spine Surgery Unit, University of Turin. “Città Della Salute e Della Scienza”-CTO Hospital of Turin, Turin, Italy

**Keywords:** length of hospital stay, spine surgery, predictive model, sociodemographic factors, patient reported outcome measures

## Abstract

**Study Design:**

Retrospective Cohort Study.

**Objectives:**

To develop and validate a multivariable predictive model for length of hospital stay (LOS) following spine surgery, incorporating sociodemographic characteristics, medical data, and self-reported patient outcomes.

**Methods:**

A retrospective analysis of 4583 patients from a spine surgery registry was conduct-ed. Predictors included age, sex, BMI, ASA score, surgical complexity, and patient-reported outcomes. Binary logistic regression was used to model LOS (<3 days vs ≥3 days).

**Results:**

Lower age, active work status, lower ASA scores, and specific surgical procedures were associated with shorter LOS. The model demonstrated good accuracy and dis-criminative ability.

**Conclusions:**

Sociodemographic, medical, and patient-reported outcomes are valuable predictors of LOS. These findings can help improve preoperative planning and resource allocation in spine surgery.

## Introduction

The length of hospital stay (LOS) following spine surgery is a critical factor influencing patient outcomes and satisfaction, health care costs, and resource utilization. Understanding the predictors of LOS is essential for optimizing patient management and improving surgical outcomes. Previous studies have identified various factors such as patient demographics, comorbidities, and surgical complexity as key determinants of LOS.^1,2^ However, the integration of sociodemographic data, medical history, and patient-reported outcomes in a predictive model remains underexplored.

Recent research has increasingly focused on the role of psychosocial factors, including pain perception and mental health, in determining postoperative recovery trajectories.^3,4^ These factors, alongside traditional clinical predictors, could provide a more comprehensive understanding of LOS determinants. Despite advancements in surgical techniques and perioperative care, there is still considerable variability in LOS among patients undergoing spine surgery, underscoring the need for predictive models that can guide preoperative planning and resource allocation.

This study aims to develop and validate a multivariable predictive model for LOS following spine surgery, incorporating sociodemographic characteristics, medical data, and self-reported patient outcomes. By identifying key predictors, this study seeks to enhance preoperative risk stratification and inform targeted interventions to reduce LOS and improve overall patient care.

## Methods

### Source of Data and Participants

This prediction study was conducted from an institutional spine surgery registry - SpineReg,^5^ with data collected in a single spine surgery center in Milan, Italy, between January 2016 and March 2022. The institutional ethics committee approved the conduct of this study (ethics committee of the San Raffaele hospital - Milan IRCCS - substantial amendment no. 3 of 05/09/2019 given the previous approval to the SPINEREG register with the number of the register of opinions of the ethics committee 93/INT/2015), and each participant gave written consent before their data joined the registry. The inclusion criteria in this study consisted of adults (>18 years old) undergoing elective spine surgery. The exclusion criteria were a diagnosis of neoplasm or infection. Additionally, individual cases were excluded when the diagnosis condition or the intervention procedure presented a representation in the registry smaller than 35 cases (ie, equivalent to 0.5% of the potential eligible cases), and they were not classifiable within a broader category according to the International Classification of Diseases (ICD).^6^ Excluded, included, and grouped ICD codes of diagnoses and procedures are presented as supplementary material (Supplemental Material 1).

This study has been reported according to the Transparent Reporting of a multivariable prediction model for Individual Prognosis or Diagnosis (TRIPOD) statement.^7^ This study followed the STROBE cohort reporting guidelines.^8^

### Outcome Measure

The primary outcome was the length of stay in the hospital expressed in terms of a binary outcome: hospital stay <3 days vs ≥3 days. Hospital stay begins the day of the surgery and ends when the patient is discharged from the hospital. This outcome was collected by the administrative staff in the surgery center.

### Predictors

The predictors can be divided into 3 categories: sociodemographic data, medical data, and self-reported questionnaires.

Sociodemographic data consisting of age, sex (ie, male or female), work status (ie, active working or not active working), body mass index (BMI), and smoking status (ie, smoker or no smoker) were collected by the administrative staff of the surgery center.

Medical data consisting of the location of the expected surgery (ie, cervical or thoraco-lumbar), number of spinal levels expected to be intervened, score in the physical status classification system according to the American Society of Anesthesiologists (ie, low-risk: ASA <3 or high risk: ASA ≥3), and ICD codes for diagnosis and procedures, and were collected by the surgeons of the surgery center.

Self-reported questionnaires evaluating disability related to the spinal condition, spinal and radicular pain intensity, quality of life, and fear-avoidance beliefs were collected by health care professionals from the surgery center. Spinal-pain-related disability was assessed with the Oswestry Disability Index (ODI)^9^ or the Neck Disability Index (NDI)^10^ for patients with a thoraco-lumbar or a cervical condition, respectively. ODI and NDI consist of 10 items rated from 0 to 5. The sum of these 10 items is expressed as a percentage, where a higher percentage score expresses higher levels of disability.^11,12^ Spinal and radicular pain intensity at rest was rated on an 11-point numeric rating scale, where 0 indicated ‘no pain’ and 10 ‘the worst imaginable pain’.^13^ Quality of life was assessed with the 36-Item Short Form Health Survey (SF-36).^14^ The SF-36 evaluates 8 domains with final values expressed as a percentage ranging from 0 (worst health) to 100 (best health). The scores in these 8 domains can be reduced to 2 general components named physical component summary (PCS) and mental component summary (MCS).^15^ The fear-avoidance beliefs about how physical activity may affect spinal pain and related disability were assessed with the Fear-Avoidance Beliefs Questionnaire (FABQ-PA). The FABQ-PA includes 4 items scored from 0 to 6 and contains, where higher scores indicate greater levels of fear-avoidance beliefs.^16^ Total scores can be divided into low-fear (FABQ-PA <15) and high-fear (FABQ-PA ≥15).^17^

All predictor variables were collected before the patient underwent surgery, in a pre-operatory evaluation session to determine the need for and characteristics of spinal surgery, in a period of 1 to 60 days prior to admission to the hospital.

### Missing Data

The number of days staying in the hospital after the surgery was missing in 1.5% of cases. Table 1 shows the rates of missing data for the predictor variables. Missing values ranged from approximately 0.0% for most of the sociodemographic and medical predictors to 16% for BMI and spinal levels expected to be treated. Additionally, approximately 3% of self-reported questionnaires were missing. Independent variables of sociodemographic data and self-reported questionnaires with at least 1 missing value were imputed using predictive mean matching for numerical variables and binary logistic regression for binary variables. Medical data variables were not imputed to avoid the introduction of bias into the results. Only patients with the observed outcome variable were included in the analysis.

**Table 1.** Percentage of Missing Values per Pre-operative Predictor Variables.

Age	Sex	BMI	Smoking status	Work status
0.0%	0.0%	16.0%	0.0%	0.0%
ASA class	Location	Spinal levels	ICD diagnosis	ICD procedure
0.0%	0.0%	15.6%	0.0%	0.0%
ODI/NDI	Spinal pain	Radicular pain	SF-36	FABQ-PA
2.7%	2.6%	2.6%	2.8%	2.9%

ASA: American Society of Anesthesiologist class; BMI: Body Mass Index; FABQ-PA: Fear-Avoidance Beliefs Questionnaire – Physical Activity subscale; ICD: International Classification of Diseases codes; NDI: Neck Disability Index; NRS: Numeric Rating Scale; ODI: Oswestry Disability Index; SF-36: 36-Item Short Form Health Survey.

### Statistical Analysis

Statistical analysis was performed using SPSS v.25 (IBM, Chicago, IL, USA), and statistical significance was set at *P* < 0.05. Variables were reported as mean and standard deviation, median and interquartile range, or n and percentage as appropriate.

Independent t-tests, Exact Fisher tests, or Chi-squared tests were conducted to compare predictor variables between patients presenting or not the outcome variable (ie, hospital stay <3 days vs ≥3 days). Bonferroni correction considering the adjusted standardized residual values was applied when a significant Chi-squared test.^18^

A binary logistic regression analysis with a Likelihood-Ratio forward method was used to predict the outcome variable: hospital stay <3 days vs ≥3 days. The goodness-of-fit of the model was evaluated with the Hosmer-Lemeshow Test. The performance of the model was evaluated in terms of sensitivity (ie, probability of a truly stay ≥3 days between those patients that truly had a stay ≥3 days), specificity (ie, probability of a truly stay <3 days between those patients that truly had a stay <3 days), positive predictive value (ie, probability of a truly stay ≥3 days between those patients classified for a stay ≥3 days), negative predictive value (ie, probability of a truly stay <3 days between those patients classified for a stay <3 days), accuracy (ie, probability of a true classification, both truly stay <3 days and truly stay ≥3 days, between all patients) and area under the receiver operating characteristic curve (AUC ROC) (ie, the average value of sensitivity for all possible values of specificity).^19^ To assess the stability and variability of the model’s coefficients, we conducted bootstrapping analysis with 1000 resamples.

## Results

A total of 4583 out of 6999 patients who underwent spinal surgery were included in this study. On the one hand, 562 patients were excluded due to age <18 years old, 39 due to cancer, 38 due to infection, and 186 because presenting a diagnosis condition or intervention procedure under-represented (ie, lower than 20 cases) in the institutional spine surgery registry (more details in Supplemental Material 1). On the other hand, 86 cases were removed from the analysis due to missing data in the outcome variable and 1505 cases due to missing data in the medical variables as predictors.

Table 2 presents the characteristics of the patients according to the predictor variables and the comparison between the patients achieving or not the outcome measure (ie, hospital stay <3 days vs ≥3 days). The patients staying in the hospital for less than 3 days presented lower age, lower proportion of females, more active workers, lower proportion of patients with an ASA score ≥3, higher rate of cervical surgery, lower number of expected spinal levels to be treated, lower spinal pain, higher radicular pain, and lower scores in the MCS of the SF-36 compared to those staying in the hospital 3 days or more. Besides, those included in an ICD diagnosis category of intervertebral disc disorders or pathological fracture, together with those where the ICD intervention procedure was categorized as excision of intervertebral discs or vertebral augmentation, had a higher proportion of staying in the hospital less than 3 days compared to those staying in the hospital 3 days or more. On the contrary, those included in an ICD diagnosis category of spondylolisthesis (degenerative and congenital), mechanical complications, or idiopathic or degenerative deformities; together with those where the ICD intervention procedure was categorized as spinal fusion or vertebral osteotomy or ostectomy had a lower proportion of staying in the hospital less than 3 days compared to those staying in the hospital 3 days or more.

**Table 2.** Descriptive Data of Predictor Variables of Participants Included in the Predictive Model and Comparisons Between Participants Staying <3 days vs ≥ 3 days After Surgery.

	All *n* = 4583	< 3 days *n* = 1316	≥ 3 days *n* = 3267	*P*
Age (years)	56.2 ± 15.2	53.1 ± 15.7	57.5 ± 14.8	<0.001
Female (n, %)	2642 (57.6%)	566 (43.0%)	2076 (63.5%)	<0.001
BMI (kg/m^2^)	25.3 ± 4.5	25.3 ± 4.3	25.3 ± 4.5	0.867
Smoker (n, %)	974 (21.3%)	276 (21.0%)	698 (21.3%)	0.780
Working (n, %)	3536 (77.2%)	1078 (81.9%)	2458 (75.2%)	<0.001
ASA ≥3 (n, %)	522 (11.4%)	95 (7.2%)	427 (13.1%)	<0.001
Location: Cervical (n, %)	353 (7.7%)	195 (14.8%)	158 (4.8%)	<0.001
Spinal levels	1 [1-2]	1 [1-2]	2 [1-3]	<0.001
ICD diagnosis (n, %)				<0.001
*Intervertebral disc disorders*	1963 (42.8)	927 (70.4%)	1036 (31.7%)	*
*Degenerative spondylolisthesis*	632 (13.8%)	6 (0.5%)	626 (19.2%)	*
*Spinal stenosis*	424 (9.3%)	176 (13.4%)	248 (7.6%)	
*Degenerative deformities*	327 (7.1%)	1 (0.1%)	326 (10%)	*
*Mechanical complication*	245 (5.3%)	17 (1.3%)	228 (7.0%)	*
*Idiopathic deformities*	222 (4.8%)	1 (0.1%)	221 (6.8%)	*
*Postoperative deformities*	219 (4.8%)	3 (0.2%)	216 (6.6%)	
*Spondylosis & allied disorders*	215 (4.7%)	90 (6.8%)	125 (3.8%)	
*Congenital spondylolisthesis*	185 (4%)	0 (0.0%)	185 (5.7%)	*
*Closed Fracture*	79 (1.7%)	39 (3%)	40 (1.2%)	
*Pathological fracture*	72 (1.6%)	56 (4.3%)	16 (0.5%)	*
ICD procedures (n, %)				<0.001
*Spinal fusion*	2448 (53.4%)	182 (13.8%)	2266 (69.4%)	*
*Excision of intervertebral disc*	936 (20.4%)	782 (59.4%)	154 (4.7%)	*
*Spinal decompression*	900 (19.6%)	238 (18.1%)	662 (20.3%)	
*Vertebral osteotomy & ostectomy*	156 (3.4%)	5 (0.4%)	151 (4.6%)	*
*Vertebral augmentation*	91 (2.0%)	89 (6.8%)	2 (0.1%)	*
*Removal of implanted device*	52 (1.1%)	20 (1.5%)	32 (1.0%)	
ODI or NDI (0-100)	46.4 ± 18.5	46.1 ± 19.7	46.5 ± 18.0	0.564
Spinal pain (0-10)	6.3 ± 3.0	5.4 ± 3.1	6.7 ± 2.8	<0.001
Radicular pain (0-10)	6.7 ± 3.2	7.1 ± 2.9	6.5 ± 2.8	<0.001
SF-36 PCS (100-0)	33.0 ± 7.5	33.2 ± 7.3	32.9 ± 7.6	0.139
SF-36 MCS (100-0)	46.3 ± 11.7	47.1 ± 11.7	46.0 ± 11.7	0.004
FABQ-PA high fear (n, %)	4104 (89.5%)	1189 (90.3%)	2915 (89.2%)	0.286

Values are expressed in mean ± standard deviation, median [interquartile range], or n (%). ASA: American Society of Anesthesiologist class; BMI: Body Mass Index; FABQ-PA: Fear-Avoidance Beliefs Questionnaire – Physical Activity subscale; ICD: International Classification of Diseases codes; MCS: Mental Component Summary; NDI: Neck Disability Index; NRS: Numeric Rating Scale; ODI: Oswestry Disability Index; PCS: Physical Component Summary; SF-36: 36-Item Short Form Health Survey. *P value <0.05 after Bonferroni correction considering the adjusted standardized residual values after Chi-squared test.

### Predicting Model

The binary logistic regression analysis with a Likelihood-Ratio forward method showed a 10 steps model including sequentially the following predictor variables: (1) ICD procedure, (2) spine location, (3) ICD diagnosis, (4) expected spinal levels to be treated, (5) ODI, (6) sex, (7) ASA class, (8) radicular pain, (9) age, (10) spinal pain. The Hosmer-Lemeshow Test showed an appropriate goodness-of-fit of the model (χ2 (8) = 9.197; *P* = 0.326). Table 3 presents the performance indicators of the model, suggesting a good to excellent performance with balanced sensitivity (92.5%) and specificity (82.2%) values, and providing a good accuracy (89.6%) with an excellent ROC AUC curve (0.954).

**Table 3.** Performance Indicators of the Predictive Model of a Hospital Stay ≥3 days.

		Observed		Total	Performance Indicators	
		≥3 days	<3 days			
Predicted	≥3 days	3026	241	3267	Sensitivity	92.6%
	<3 days	234	1082	1316	Specificity	82.2%
	Total	3260	1323	4583	PPV	92.8%
					NPV	81.8%
					Accuracy	89.6%
					AUC ROC	0.954

PPV: Positive Predictive Values; NPV: Negative Predictive Values.

A summary of the included variables and bootstrapping analysis are presented in the Supplemental Material.

## Discussion

The results of this study underscore the multifactorial nature of predicting the length of hospital stay after spine surgery, integrating sociodemographic, medical, and self-reported factors. The predictive model demonstrated that variables such as the type of surgical procedure, spinal location, expected number of spinal levels, ASA classification, and self-reported pain and disability scores significantly influence the duration of hospitalization. This discussion will explore the implications of these findings, comparing them with existing literature and discussing their potential impact on clinical practice.

### Surgical and Medical Predictors

The type of surgical procedure and the spinal location were among the strongest predictors of hospital stay duration. Specifically, patients undergoing procedures categorized as “excision of intervertebral discs” or “vertebral augmentation” had shorter hospital stays, likely due to the less invasive nature and quicker recovery times associated with these surgeries. In contrast, more complex procedures, such as spinal fusion or vertebral osteotomy, were associated with longer stays, reflecting the increased recovery time required for these extensive surgeries. These findings align with previous research, which has consistently shown that the complexity and invasiveness of spinal procedures are major determinants of postoperative recovery and length of stay.^2,20^

The ASA classification, which assesses the physical status of patients and their pre-operative comorbidities, was another significant predictor. Patients classified as ASA ≥3, indicating higher surgical risk, had longer hospital stays. This is consistent with the broader literature that highlights the ASA score as a reliable predictor of post-operative outcomes, including complications and recovery time.^21,22^ The association between higher ASA scores and longer stays underscores the importance of thorough preoperative evaluation and optimization of comorbid conditions to potentially reduce hospital stays.

### Sociodemographic Predictors

Among the sociodemographic factors, age and work status were significant predictors. Older age was associated with longer hospital stays, which is in line with numerous studies that have demonstrated age as a critical factor influencing recovery. Older patients often have multiple comorbidities, reduced physiological reserve, and slower recovery processes, all of which contribute to prolonged hospitalizations.^23^ Additionally, patients who were actively working prior to surgery had shorter hospital stays, possibly reflecting better overall health and physical condition, which facilitate quicker recovery.

### Self-Reported Measures

Self-reported pain and disability scores also played a crucial role in predicting hospital stay. Higher scores on the Oswestry Disability Index (ODI) and Neck Disability Index (NDI), indicating greater disability, were associated with longer hospital stays. Similarly, higher preoperative pain levels, particularly spinal pain, were predictors of extended hospitalization. These findings are consistent with existing research that emphasizes the prognostic value of preoperative pain and disability levels in predicting postoperative recovery.^4,24^

Interestingly, the mental component summary (MCS) of the SF-36, a measure of mental health, also emerged as a significant predictor. Patients with lower MCS scores, indicating poorer mental health, had longer hospital stays. This highlights the role of psychological factors in recovery from surgery, with mental health potentially influencing pain perception, motivation for recovery, and overall resilience. The impact of psychological factors on postoperative outcomes has been increasingly recognized, with studies suggesting that interventions aimed at improving mental health could potentially shorten recovery times and reduce hospital stays.^3^

### Integration with AI-Driven Optimization of PROMs Registries

To further enhance the precision and applicability of predictive models, it is essential to consider the role of Patient Reported Outcome Measures (PROMs) registries. PROMs provide a patient-centered approach by capturing subjective assessments of clinical conditions, disability, and quality of life, which are crucial for monitoring changes over time and evaluating the effectiveness of treatments from the patient’s perspective.

However, 1 key challenge with PROMs-based registries is the low response rate, often due to the effort required by patients to complete lengthy or complex questionnaires. Integrating AI-based technologies can address this challenge by making the questionnaires more interactive and engaging, improving patient engagement and data quality. For instance, AI algorithms can predict patient non-compliance and adapt the questionnaires to individual patient needs, reducing the effort required and enhancing the overall completion rate.

These innovations are directly applicable to spine surgery, where accurate preoperative risk stratification and efficient resource allocation are critical. By leveraging AI-driven optimisation of PROMs data collection, health care providers can obtain more reliable data that enhances predictive accuracy, supports clinical decision-making, and ultimately leads to improved patient care outcomes.

This approach aligns with the broader goal of enhancing patient engagement and optimising data acquisition, which is crucial for advancing evidence-based practices in musculoskeletal health care. Thus, incorporating AI and machine learning into PROMs registries not only addresses data collection challenges but also maximises the potential for predictive insights that can significantly impact clinical outcomes and patient management strategies.^25^

### Clinical Implications

The findings from this study have important implications for clinical practice. Under-standing the predictors of hospital stay can assist clinicians in preoperative planning and patient counseling. For instance, identifying patients at risk for longer hospitalizations allows for targeted interventions, such as rehabilitation programs, optimization of comorbidities, and enhanced postoperative care plans. Moreover, this in-formation can aid in resource allocation and scheduling, particularly in high-volume spine surgery centers where efficient bed management is crucial.

**Figure 1.**
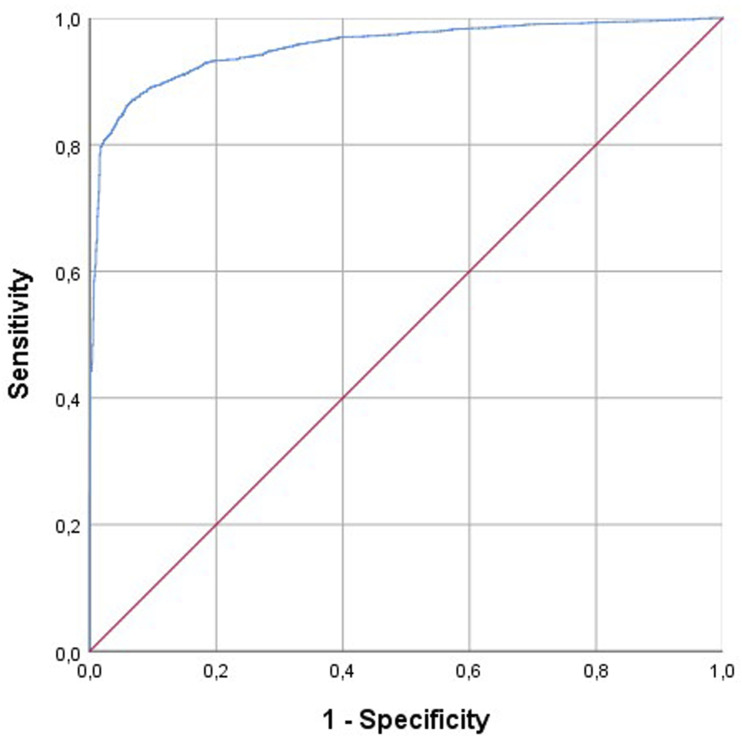
ROC Curve for the model predicting a stay in the hospital lower than 3 days.

The predictive model developed in this study, with an AUC of 0.954, indicates excel-lent discriminative ability, suggesting that it could be a valuable tool in clinical set-tings (Figure 1). However, the application of this model requires validation in other populations and settings to ensure its generalizability. Additionally, while the model incorporates a wide range of predictors, future research could explore the inclusion of other factors, such as socioeconomic status, access to health care resources, and support systems, which might also influence hospital stay duration.

### Limitation and Future Directions

This study has several limitations that should be acknowledged. First, it was conducted at a single center, which may limit the generalizability of the findings. Multi-center studies are needed to validate the predictive model and assess its applicability across different health care settings. Second, while the study included a comprehensive set of predictors, there may be other unmeasured variables that influence hospital stay, such as intraoperative factors and postoperative complications. Future research should aim to include these variables to develop a more robust predictive model. Third, model performance was evaluated on the training dataset, which may lead to overfitting and reduced generalizability. However, the stability of key coefficients after bootstrapping suggests that our model reliably captures associations between predictors and outcomes, reinforcing its potential clinical relevance. Accordingly, future studies should incorporate external validation or cross-validation to further confirm its robustness. Moreover, both cervical and thoracolumbar surgeries were included: this can represent a bias because of the intrinsic differences between these regions.

In conclusion, this study identifies key predictors of hospital stay duration after spine surgery, providing valuable insights for preoperative assessment and planning. By integrating these predictors into clinical practice, health care providers can improve patient outcomes, optimize resource utilization, and enhance the overall efficiency of spine surgery care pathways.

## Supplemental Material

Supplemental Material - Identifying Key Factors Influencing Hospital Stay After Spine Surgery: A Comprehensive Predictive Model Supplemental Material for Identifying Key Factors Influencing Hospital Stay After Spine Surgery: A Comprehensive Predictive Model by Francesco Langella, Francesca Barile, Pablo Bellosta-Lòpez, Federico Fusini, Domenico Compagnone, Daniele Vanni, Marco Damilano, and Pedro Berjano in Global Spine Journal.

## Data Availability

The datasets used and/or analyzed in the present study are available from the corresponding author upon reasonable request.*
