# Supplementary material for: Identifying Key Factors Influencing Hospital Stay After Spine Surgery: A Comprehensive Predictive Model
Source: Global Spine J. 2025 Apr 1;15(8):3756–63. doi: 10.1177/21925682251331451 (PMC11962937; doi:10.1177/21925682251331451)
Supplement: Supplemental Material - Identifying Key Factors Influencing Hospital Stay After Spine Surgery: A Comprehensive Predictive Model [file sj-pdf-1-gsj-10.1177_21925682251331451.pdf]

Supplementary Table. Logistic Regression Results with Bootstrapped Validation: Coefficients, Odds Ratios, and Confidence Intervals.

Variables	B (Log-Odds)	Error Standard	Wald	Sig.	Exp(B)	95% C.I. for Exp(B)	B (Bootstrapping)	Bias	Error Standard (Bootstrapping)	Sig. (Bootstrapping)	95% C.I. (Bootstrapping)
**Female**	-0.488	0.113	18.542	<0.001	0.614	0.491 - 0.766	-0.488	-0.016	0.112	<0.001	-0.709 to -0.280
**Age**	0.013	0.004	9.437	0.002	1.013	1.005 - 1.022	0.013	0.000	0.004	0.002	0.005 to 0.021
**ODI or NDI**	0.015	0.003	20.134	<0.001	1.016	1.009 - 1.023	0.015	0.000	0.004	<0.001	0.009 to 0.023
**Spinal pain**	0.051	0.019	7.358	0.007	1.053	1.014 - 1.092	0.051	0.000	0.019	0.007	0.014 to 0.091
**Radicular pain**	-0.093	0.023	16.909	<0.001	0.911	0.872 - 0.953	-0.093	-0.001	0.023	<0.001	-0.138 to -0.049
**Location: cervical**	-3.208	0.222	209.483	<0.001	0.040	0.026 - 0.062	-3.208	-0.072	0.266	<0.001	-3.837 to -2.791
**ASA ≥3**	-0.630	0.193	10.629	0.001	0.532	0.364 - 0.778	-0.630	-0.009	0.216	0.005	-1.075 to -0.225
**Spinal levels**	0.164	0.033	23.960	<0.001	1.178	1.103 - 1.258	0.164	0.040	0.110	0.026	0.087 to 0.555
**ICD diagnosis (1)**	-0.179	0.224	0.639	0.424	0.836	0.540 - 1.296	-0.179	0.005	0.264	0.470	-0.726 to 0.337
**ICD diagnosis (2)**	-0.982	0.168	34.111	<0.001	0.374	0.269 - 0.521	-0.982	-0.024	0.188	<0.001	-1.394 to -0.661
**ICD diagnosis (3)**	-1.721	0.742	5.384	0.020	0.179	0.042 - 0.765	-1.721	0.499	3.049	0.048	-3.338 to 2.499
**ICD diagnosis (4)**	1.174	1.135	1.071	0.301	3.236	0.350 - 29.918	1.174	5.656	7.439	0.059	-0.111 to 17.090
**ICD diagnosis (5)**	2.012	0.700	8.254	0.004	7.475	1.895 - 29.485	2.012	0.914	3.697	0.005	0.817 to 18.370
**ICD diagnosis (6)**	2.882	1.076	7.174	0.007	17.854	2.167 - 147.126	2.882	5.447	7.600	0.019	1.186 to 18.598
**ICD diagnosis (7)**	3.140	0.435	52.111	<0.001	23.105	9.850 - 54.197	3.140	0.142	1.044	<0.001	2.383 to 4.516
**ICD diagnosis (8)**	19.789	2688.324	0.000	0.994	>100	-	19.789	-0.031	0.353	<0.001	18.999 to 20.396
**ICD diagnosis (9)**	0.390	0.721	0.292	0.589	1.476	0.360 - 6.061	0.390	0.720	3.333	0.680	-1.372 to 18.096
**ICD diagnosis (10)**	0.698	0.483	2.093	0.148	2.010	0.781 - 5.178	0.698	0.078	0.569	0.179	-0.195 to 2.075
**ICD procedure (1)**	2.604	0.159	269.212	<0.001	13.514	9.902 - 18.444	2.604	0.023	0.174	<0.001	2.293 to 2.973
**ICD procedure (2)**	5.160	0.544	89.940	<0.001	174.244	59.977 - 506.211	5.160	0.335	1.773	<0.001	4.271 to 7.024
**ICD procedure (3)**	0.259	0.526	0.243	0.622	1.296	0.462 - 3.634	0.259	-0.092	0.687	0.679	-1.348 to 1.403
**ICD procedure (4)**	4.740	0.199	567.626	<0.001	114.417	77.472 - 168.979	4.740	0.059	0.203	<0.001	4.410 to 5.243
**ICD procedure (5)**	-3.778	1.092	11.961	0.013	0.023	0.003 - 0.195	-3.778	-3.988	8.354	0.013	-29.688 to -1.672
**Constant**	-2.056	0.401	26.331	<0.001	0.128	-	-2.056	-0.053	0.396	<0.001	-2.835 to -1.314

“ICD diagnosis” includes ten categories, with “Intervertebral disc disorders” serving as the reference category. The other categories are: (1) Spondylosis & allied disorders, (2) Spinal stenosis, (3) Pathological fracture, (4) Idiopathic deformities, (5) Postoperative deformities, (6) Degenerative deformities, (7) Degenerative spondylolisthesis, (8) Congenital spondylolisthesis, (9) Closed fracture of the vertebral column without mention of spinal cord lesion, and (10) Mechanical complication.

“ICD procedures” includes five categories, with “Excision of intervertebral disc” serving as the reference category. The other categories are: (1) Spinal decompression, (2) Vertebral osteotomy & ostectomy, (3) Removal of implanted device, (4) Spinal fusion, and (5) Excision of intervertebral disc.

Supplementary material 1. Number of cases per ICD codes included, excluded, and grouped.

- ICD-diagnosis codes selected:

SPONDYLOSIS AND ALLIED DISORDERS	
code	n
721	20
721.1	160
721.2	2
721.3	42
721.41	4
721.42	29
721.6	2
721.9	2
721.91	1

INTERVERTEBRAL DISC DISORDERS	

code	n
722	104
722.1	1143
722.11	6
722.2	5
722.4	21
722.51	5
722.52	842
722.6	64
722.7	1
722.71	91
722.72	8
722.73	28
722.8	1
722.83	31
722.9	1
722.92	1
722.93	1

SPINAL STENOSIS	
code	n
723	15
724.01	4
724.02	533
724.09	2

PATHOLOGICAL FRACTURE	
code	n
733.1	35
733.13	84

IDIOPATHIC DEFORMITIES	
code	n
737	6
737.3	72

737.31	1
737.32	174

POSTOPERATIVE DEFORMITIES	
code	n
737.1	28
737.12	24
737.19	238
737.22	5

DEGENERATIVE DEFORMITIES	
code	n
737.39	320
737.4	1
737.41	9
737.43	27
738.5	58

DEGENERATIVE SPONDYLOLISTHESIS	
code	n
738.4	741

CONGENITAL SPONDYLOLISTHESIS	
code	n
756.12	212

CLOSED FRACTURE OF VERTEBRAL COLUMN WITHOUT MENTION OF SPINAL CORD LESION	
code	n
805	2
805.02	2
805.07	1

805.2	25
805.4	54
805.6	1
805.8	18

MECHANICAL COMPLICATION	
code	n
996.4	110
996.49	204
996.59	1
996.78	28

- ICD-diagnosis codes excluded due to neoplasm or infection:

NEOPLASM	
code	n
170.2	5
198.5	5

203	2
213.2	6
225.3	7
225.4	4
225.8	2
225.9	4
228	1
237.71	3

<u>INFECTION</u>

code	n
996.66	2
996.67	11
996.69	1
998.32	2
998.51	2
998.59	17
998.6	2

998.83	1

- ICD-diagnosis codes excluded due to low representation (<35 cases) and not classifiable within a broader category:

code	n
332	1
336	1
336.3	2
336.8	2
343.8	1
344	1
349.2	2
350.1	1
353.4	1
388.5	1
602.8	1
715.15	1
718.28	1
720.2	3
723.2	1

724.2	2
724.3	1
724.4	2
724.5	4
724.79	5
724.9	8
727.4	14
727.89	1
730.18	1
730.28	1
730.9	1
730.98	2
732	1
732.8	2
733.29	1
733.82	20
733.95	1
754.2	4

756.11	24
756.51	1
805.9	1
806.2	1
806.4	3
839.01	1
839.41	2
905.1	7
996.2	1
996.3	2
996.39	1
998.89	2

- ICD-procedures codes selected:

SPINAL DECOMPRESSION	
code	n
3,02	12

3,09	1098

VERTEBRAL OSTEOTOMY & OSTECTOMY

code	n
77,09	1
77,29	12
77,79	54
77,8	1
77,89	20
77,9	1
77,99	7
78	12
78,09	90
78,4	1
78,49	18

REMOVAL OF

IMPLANTED DEVICES FROM VERTEBRAE	
code	n
78,6	9
78,69	101

EXCISION OF INTERVERTEBRAL DISC	
code	n
80,51	1117

PERCUTANEOUS VERTEBROPLASTY & VERTEBRAL AUGMENTATION	
code	n
81,65	134
81,66	3

SPINAL FUSION	
code	n
81	1
81,01	5
81,02	270
81,03	23
81,04	18
81,05	496
81,06	440
81,07	18
81,08	1229
81,3	1
81,32	1
81,33	1
81,35	31
81,36	4
81,38	42

81,62	178
81,63	59
81,64	114
84,51	238
84,55	1
84,59	6

- ICD-procedures codes excluded due to low representation (<35 cases) and/or not classifiable within a broader category:

OPERATIONS ON SPINAL CORD STRUCTURES	
code	n
3.4	18
3.52	1
3.59	4
3.6	1

INFECTION REMOVAL	

code	n
86.22	25

code	n
0.31	4
1.25	1
2.11	1
2.12	1
3.32	3
3.93	1
3.99	2
4.07	2
4.41	1
34.01	2
54.11	1
59.8	1
76.39	1
77.4	1

code	n
77.49	7
77.6	1
77.69	1
78.3	2
78.59	3
79.39	1
80.09	2
80.39	1
80.59	1
80.8	1
80.99	4
81.51	1
81.99	1
83.21	1
84.6	1
84.66	1
86.04	1

code	n
86.09	1
86.74	1
91.59	1
91.73	1
93.29	1
97.16	1
99.04	4
